# Outcome of endovascular treatment of internal iliac artery aneurysms: a single center retrospective review

**DOI:** 10.1186/s42155-022-00330-1

**Published:** 2022-10-18

**Authors:** Jinoo Kim, Joo-Young Chun, Seyed Ameli-Renani, Lakshmi Ratnam, Leto Mailli, Vyzantios Pavlidis, Raj Das, Robert Morgan

**Affiliations:** 1 Department of Radiology, St George’s University Hospitals NHS Foundation Trust, Tooting, London, UK; 2 Department of Radiology, Ajou University Hospital, Suwon, Gyeonggi-do Republic of Korea

**Keywords:** Internal iliac artery, Hypogastric artery, Aneurysm, Embolization, Vascular plug, Stent-graft

## Abstract

**Purpose:**

To evaluate the technical feasibility and clinical outcomes of endovascular treatment for internal iliac artery (IIA) aneurysms.

**Material and methods:**

This was a retrospective analysis of 25 patients with 32 IIA aneurysms (mean diameter: 39.1 ± 12.6 mm) who underwent endovascular treatment over a 10-year period, and were available for follow-up. Univariate analysis was used to determine the association between variables (including aortoiliac involvement and technique) and outcome.

**Results:**

The IIA inflow was covered with an iliac stent graft (*N* = 29) or embolized with a plug (*N* = 3). The IIA outflow was embolized in all but one case in which there was thrombotic occlusion of outflow branches. Outflow embolization using plugs or coils was performed in the distal IIA or anterior/posterior trunks in 9 cases and distal IIA branches in 22 cases. During a mean follow-up period of 39.9 months, 31.2% of aneurysms demonstrated endoleak and 12.5% demonstrated enlargement. Univariate analysis revealed that endoleak was associated with technical failure (*p* = 0.01) and that endoleak rate was higher in patients who underwent distal IIA branch embolization (*p* = 0.03). No variable was associated with sac expansion. Major complication occurred in one patient who died from aneurysm rupture. Minor complications were reported in six patients who developed femoral pseudoaneurysm (*N* = 2, 8%), buttock claudication (*N* = 3, 12%), and limb graft occlusion (*N* = 1, 4%).

**Conclusion:**

Endovascular treatment of IIA aneurysms effectively prevents sac expansion. Endoleak was more frequently observed in cases of technical failure and those in which distal IIA branches were embolized.

**Level of Evidence:**

Level 3b, retrospective cohort study.

## Introduction

Internal iliac artery (IIA) aneurysms, defined as a two-fold increase in the size of the internal iliac artery, account for 20—25% of iliac aneurysms and 0.7% of all intra-abdominal aneurysms (Muradi et al. 2014). Iliac artery aneurysms rarely occur in isolation and are commonly associated with abdominal aortic aneurysms (Huang et al. 2008). Although their natural history is poorly understood, IIA aneurysms have been associated with a rupture rate of 40% and a mortality rate of 80% (Domoto et al. 2019; Joviliano et al. 2019; Dix et al. 2005). The high mortality rate may be due to their deep location in the pelvis, where IIA aneurysms seldomly demonstrate clinical symptoms until they reach a significant size (Rana et al. 2014). However, incidental detection of IIA aneurysms has become common owing to the widespread use of cross-sectional imaging. There is a general agreement that iliac artery aneurysms exceeding 3 cm in diameter should be electively treated to prevent rupture (Muradi et al. 2014; Gao et al. 2018). Endovascular treatment is a safer alternative to open repair, the latter of which has been associated with a high perioperative mortality rate (Domoto et al. 2019; Kawatani and Oguri 2020; Machado et al. 2016). With a limited number of publications on the subject, this study aims to assess the technical feasibility of using stent-graft and vascular plug or coils for the treatment for IIAA, and to determine variables associated with treatment outcome.

## Material and methods

### Study design and population

For this retrospective study, consecutive cases of endovascular treatment for IIA aneurysms between January 2010 – December 2020 were retrieved from the electronic database at St George’s University Hospitals NHS Foundation Trust. Excluding four patients who did not undergo imaging surveillance, a total of 32 IIA aneurysms (mean diameter: 39.1 ± 12.6 mm, range: 23 – 84 mm) in 25 patients (male: female = 24: 1) were available for review. The mean age of the group was 74.9 ± 5.6 years (range, 65—89 years). One patient presented with abdominal pain, while the remainder were asymptomatic.

### Aneurysm characteristics

None of the aneurysms were ruptured on initial presentation. Twenty IIA aneurysms were associated with concomitant aortoiliac aneurysms. Two IIA aneurysms were remnant lesions left untreated during previous endovascular repair for aortic aneurysms. The remaining ten aneurysms were isolated IIA aneurysms. Baseline demographics of the patients are presented in Table 1. Technical details of the endovascular procedure were evaluated by reviewing procedure reports and angiographic images on the picture archiving and communication system.

**Table 1:**
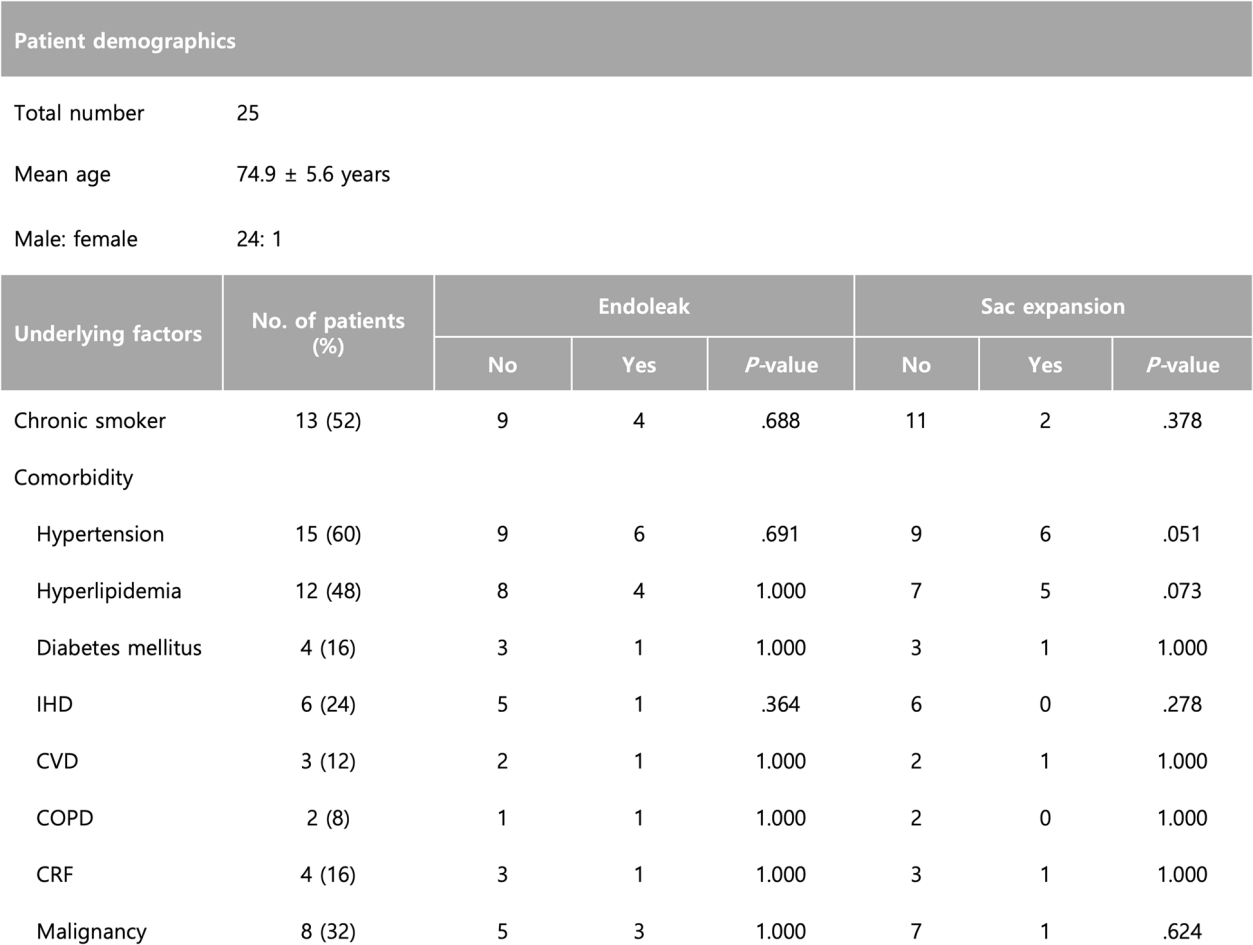
Patient demographics

*Abbreviations*: *IHD* Ischemic heart disease, *CVD* Cerebrovascular disease, *COPD* Chronic obstructive pulmonary disease, *CRF* Chronic renal failure

### Endovascular procedure

**Fig. 1:**
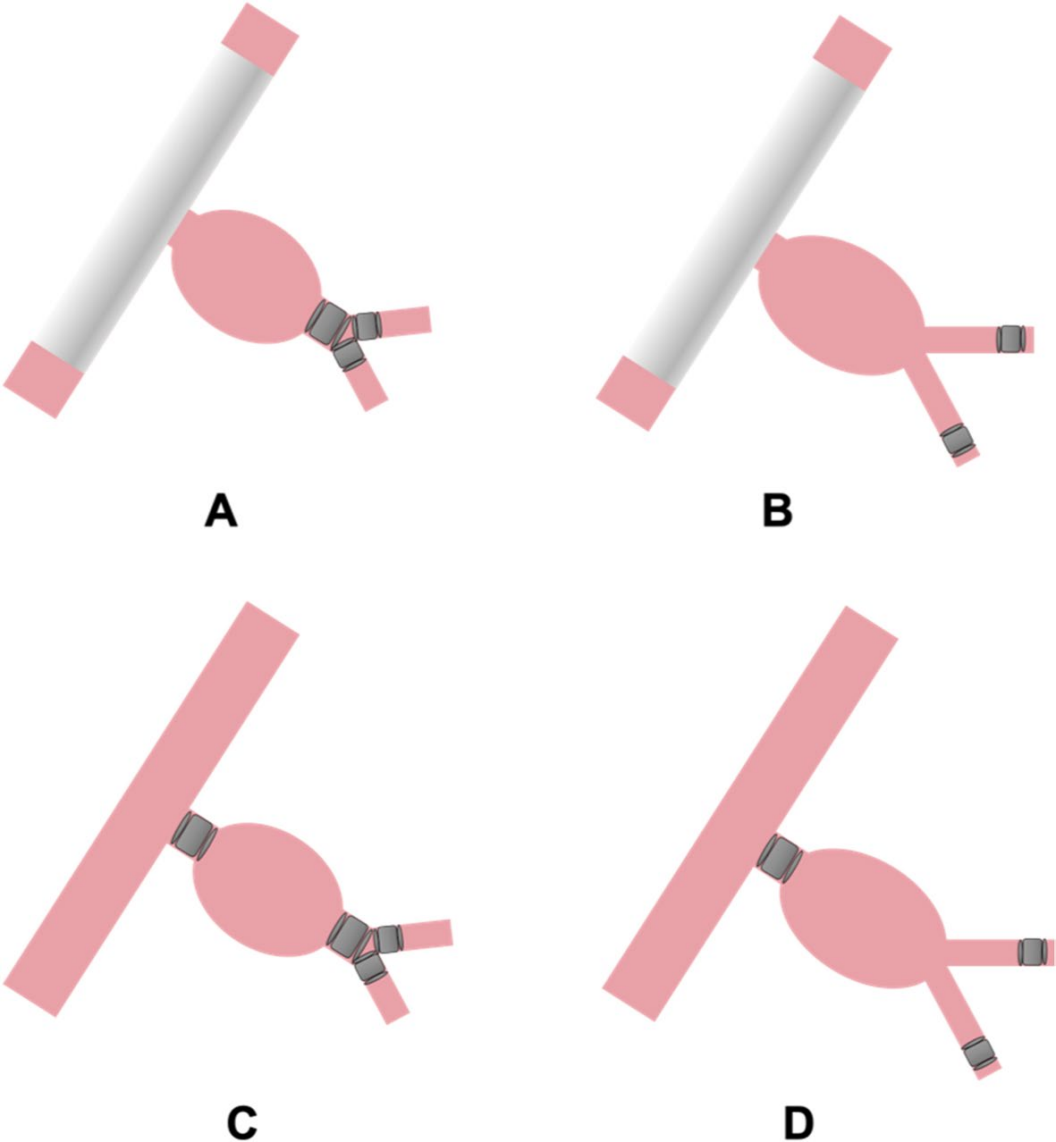
Type of endovascular technique. **A** and **B** Iliac stent graft deployed over the ostium of the IIA following outflow embolization. **C** and **D** Embolization of the inflow and outflow of the IIA. **A** and **C** Outflow embolization by placing embolic materials in the distal segment of the IIA or its bifurcation. **B** and **D** Outflow embolization by placing embolic materials in the distal branches beyond the bifurcation of the anterior and posterior trunks

**Fig. 2:**
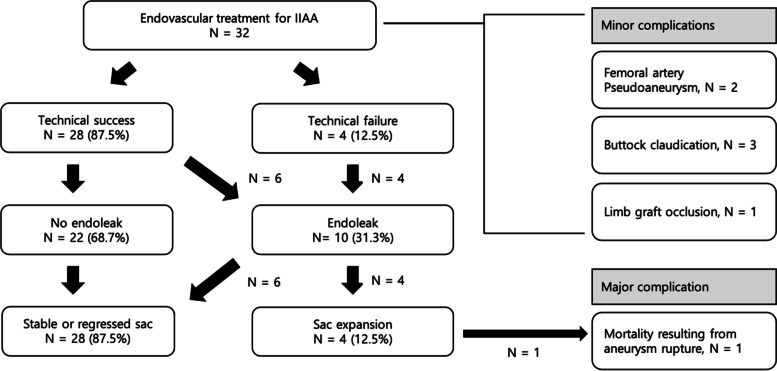
Summary of clinical outcome

All procedures were performed by interventional radiologists at St George’s University Hospitals NHS Foundation Trust with between 3 and 30 years of experience. One of the following endovascular techniques was used to exclude the IIA aneurysm: (1) stent graft deployment across the ostium of the IIA following outflow embolization (Fig. 1A and B), or (2) embolization of inflow and outflow of the IIA (Fig. 1C and D). Vascular plugs (Amplatzer Vascular Plug II or IV, Abbott Laboratories, Min, USA) or coils were used for embolization. The stent grafts and plugs were sized according to the diameters of the native arteries with 20–30% oversizing. The choice of endovascular technique and embolic material was left to the discretion of the interventional radiologist. Outflow embolization sites were categorized into the following based on the anatomical location of embolic materials: (1) embolization in the distal segment of the IIA or the anterior/posterior trunk of the IIA (Fig. 1A and C), or (2) embolization in the distal IIA branches (Fig. 1B and D).

### Outcome and follow-up

Technical success was defined as successful catheterization and interruption of blood flow in all intended target vessels. Any procedure that resulted in catheterization failure of any target vessel was categorized as a technical failure. Clinical outcome was determined by reviewing in-hospital and outpatient documents and referral letters. Procedure-related complications, re-intervention, and mortality following endovascular treatment were assessed. Complications were classified as minor or major according to the Society of Interventional Radiology clinical practice guidelines (Sacks et al. 2003). Duplex ultrasound exams and computed tomography (CT) images were analyzed for the presence of endoleak or enlargement of the IIA aneurysm.

### Statistical analysis

Categorical variables are reported as numbers and percentages, and continuous variables are presented as mean ± standard deviation values. Variables included the diameter of the IIA aneurysm, concomitant aortoiliac aneurysm, and technical details of the endovascular procedure. Univariate analysis comparing patients with endoleak or sac expansion to those without was performed using the Fisher exact test for discrete variables and Mann–Whitney U test for non-parametric, continuous variables. A *p*-value of < 0.05 was considered significant. Statistical analysis was performed using SPSS 13.0 software (SPSS, Chicago, IL, USA).

## Results

Technical and clinical outcomes are summarized in Table 2 and Fig. 2. An iliac stent graft was deployed across the IIA ostium in 29 cases, and a vascular plug was deployed in 3 cases to interrupt inflow to the IIA aneurysm. Outflow embolization was performed in the distal segment of the IIA or the anterior/posterior trunks in 9 cases, while distal IIA branches were embolized in 22. In one case, outflow embolization was not required due to chronic thrombotic occlusion of the IIA outflow. Embolic materials used for outflow embolization were coils (12 cases), plugs (10 cases), or a combination of both (10 cases). Technical success was achieved in 28 IIA aneurysms (87.5%). Four cases of technical failure resulted from unsuccessful attempts to catheterize a distal branch of the IIA in 3 patients and a misplaced vascular plug in the distal segment of the IIA in one patient.

**Table 2:**
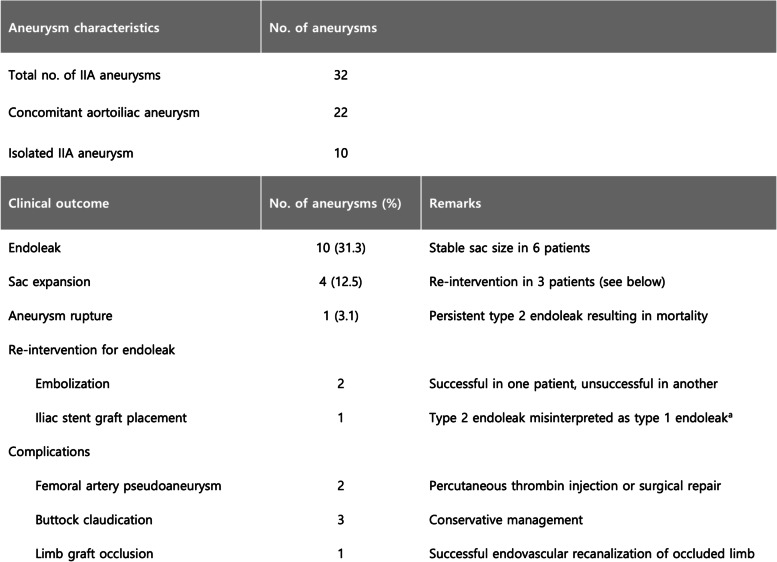
Anatomical characteristics of IIAA and association with clinical outcome

^a^A stent graft was deployed in the iliac artery to cover the ostium of the left internal iliac artery which had previously been embolized with a vascular plug

**Fig. 3:**
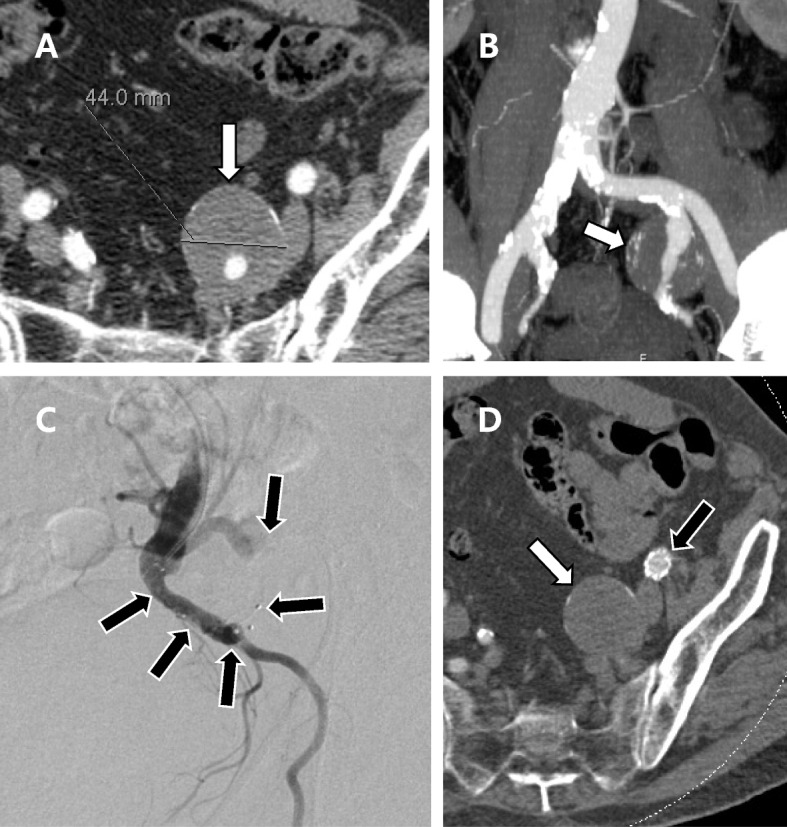
Successful endovascular treatment of an isolated IIA aneurysm in a 72-year-old, male patient. **A** and **B** CT scan demonstrates an IIA aneurysm measuring 44 mm in diameter (white arrow). **C** Vascular plugs (black arrows) were deployed in the outflow branches. The ostium of the internal iliac artery was covered by deploying an iliac stent graft (not shown). **D** CT at 3 months follow-up demonstrates complete thrombosis of the aneurysm sac (white arrow) without endoleak. Patency of the iliac stent-graft (black arrow) is maintained

All patients underwent surveillance with Duplex scans. Follow-up CT scans were also available in all but four patients. During a mean follow-up period of 39.9 months (range: 2 – 93 months), 22 IIA aneurysms (68.8%) were free from endoleak (Fig. 3). Meanwhile, type 2 endoleak was detected in 10 cases. Four of these cases were associated with technical failure. In five IIA aneurysms, follow-up CT scans demonstrated an endoleak from the iliolumbar, lateral sacral, or obturator arteries. These vessels were located proximal to the embolization sites. A final case of endoleak was located at the level of the abdominal aorta and not around the treated IIA. However, the diameter of the aortoiliac aneurysm, including the IIA aneurysm, progressively increased in size during follow-up.

**Fig. 4:**
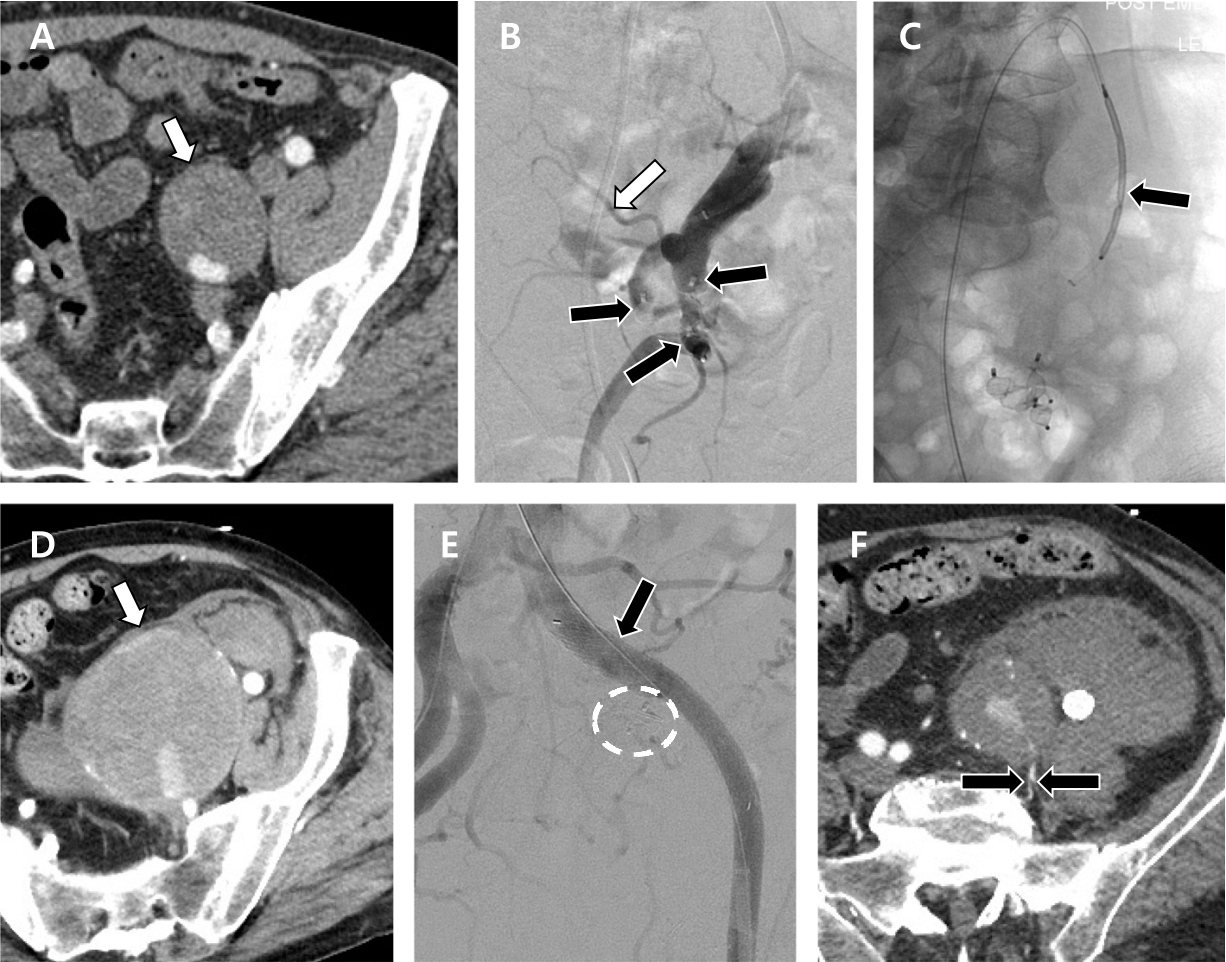
Ruptured IIA aneurysm in a 63-year-old male resulting from persistent type 2 endoleak. **A** Initial CT demonstrates an isolated IIA aneurysm (white arrow) measuring 42 mm in diameter. **B** Vascular plugs (black arrows) were placed distally to the bifurcation of the anterior and posterior trunks of the IIA. Not a prominent iliolumbar artery (white arrow) arising proximally to the embolization site. **C** A vascular plug (black arrow) was placed in the proximal segment of the IIA to interrupt the inflow. **D** The patient returned 5 years later with a ruptured aneurysm (white arrow). **E** An iliac stent-graft was deployed over the ostium of the IIA (black arrow) which had previously been embolized with a vascular plug (white circle). **F** Follow up CT acquired one week later demonstrates persistent endoleak associated with retrograde flow through a patent iliolumbar artery (located between two black arrows). The patient expired three weeks later from multiorgan failure

Sac expansion was demonstrated in 4 IIA aneurysms (12.5%) with endoleak. Re-intervention was performed in three of these cases. The patient with type 2 endoleak at the level of the abdominal aorta was successfully treated by transcatheter embolization. In another case of type 2 endoleak, transcatheter embolization was unsuccessful due to failed attempts to catheterize the feeding artery. In a third case, progressive expansion of an IIA aneurysm treated by embolization of inflow and outflow resulted in aneurysm rupture (Fig. 4). The patient underwent re-intervention where an iliac stent graft was deployed over the pre-existing vascular plug in the proximal IIA. However, the patient did not recover from the consequences of hypotensive shock and expired within three weeks with rapid progression to multiorgan failure. This patient accounted for the only case of mortality. A retrospective review of CT images suggested a type 2 endoleak associated with backflow through the iliolumbar artery.

The afore-mentioned case of mortality associated with aneurysm rupture accounted for the only case of major complication (4%). Minor complications were reported in six patients. Two patients developed femoral artery pseudoaneurysms, both associated with the percutaneous placement of stent grafts. One of the pseudoaneurysms was successfully managed by percutaneous thrombin injection and the other by surgical repair. Three patients developed buttock claudication after unilateral IIA embolization. The level of outflow embolization was in the distal IIA branches in two patients and in the distal segment of the IIA in one patient. Limb graft occlusion occurred in one patient who underwent EVAR for an aortoiliac aneurysm with IIA involvement. The occluded limb graft was successfully recanalized by percutaneous thrombectomy and stenting.

Univariate analysis revealed that technical failure was associated with the development of endoleak but not with sac expansion (Table 3). Furthermore, the outflow embolization site was associated with endoleak development but not sac expansion. A higher incidence of endoleak was demonstrated in cases where outflow embolization was performed in the distal branches of the IIA compared to when it was performed in the IIA or anterior/posterior trunks. The size of the IIA aneurysm, its association with aortoiliac aneurysms, and the type of embolic material were not associated with endoleak or sac expansion. None of the demographic factors were associated with clinical outcome.

**Table 3:**
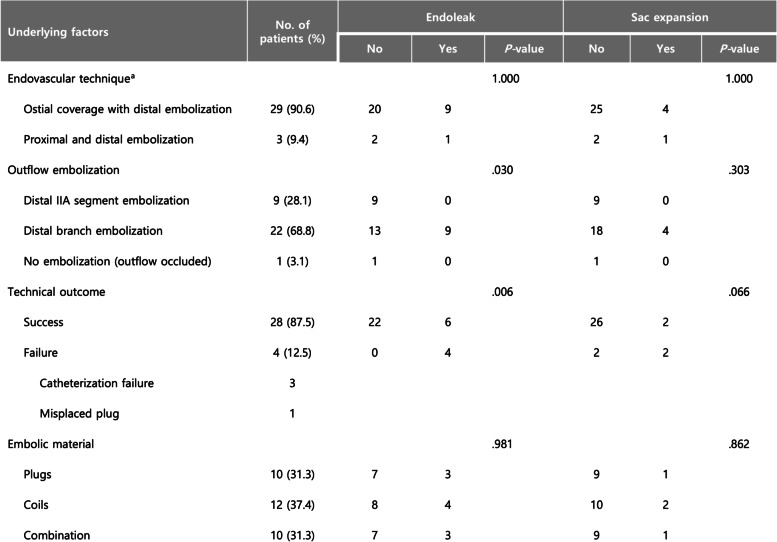
Details of endovascular treatment and association with clinical outcome

^a^Excluding re-intervention

## Discussion

The results from this study support the findings from previous publications that endovascular treatment of IIA aneurysms is effective in preventing aneurysm expansion and rupture (Rana et al. 2014; Gao et al. 2018; Machado et al. 2016; Yang et al. 2020; Chen et al. 2021; Chaer et al. 2008; Kliewer et al. 2019). The IIA is aneurysmal when its diameter exceeds 15—18 mm, and the general threshold for repair is 3 cm (Muradi et al. 2014; Kliewer et al. 2019). However, one study reported a low rupture rate for IIA aneurysms under 4 cm and recommended a conservative approach for aneurysms under this threshold (Laine et al. 2017). Others have suggested that IIA aneurysms should be treated regardless of size (Dix et al. 2005; Melki et al. 2001). Such disagreement likely results from a poor understanding of the natural history of IIA aneurysms (Dix et al. 2005; Richardson and Greenfield 1988; Wilhelm et al. 2014). In the current study, the diameter of IIA aneurysms ranged from 23 – 84 mm. Those aneurysms under 30 mm diameter were treated during the process of EVAR for aortoiliac aneurysms. Without treatment, these IIA aneurysms would have been inaccessible to further endovascular treatment after EVAR in the event of aneurysm growth.

Ten isolated IIA aneurysms were included in this study. Isolated IIA aneurysm without aortic or common iliac involvement is uncommon (Rana et al. 2014; Chen et al. 2021; Antoniou et al. 2011). Yang et al. reported the largest patient group for isolated IIAA treated by either surgical or endovascular techniques, in which forty-two patients were included (Yang et al. 2020). A recent systematic review disclosed 202 isolated IIA aneurysms from all publications, reflecting the rarity of this condition (Perini et al. 2021). Endovascular treatment for isolated IIA aneurysms can be restricted to the IIA if there is sufficient neck length in the proximal IIA to place a vascular plug or coils. When the proximal neck length is insufficient, a stent graft may be deployed in the ipsilateral iliac artery to cover the ostium of the IIA. In either of these techniques, the outflow of the IIA aneurysm should be embolized beforehand to prevent backflow. An alternative endovascular technique, which was not applied in this cohort, involves the use of a branched stent graft, or iliac bifurcation device (IBD). IBD is an appealing option in treating aortoiliac aneurysms because the blood flow in the IIA is preserved (Rana et al. 2014; Lin et al. 2009; Noel-Lamy et al. 2015; D’Oria et al. 2020). However, the use of IBD for IIA aneurysms is controversial because the presence of IIA aneurysms has been associated with technical failure during endovascular treatment of aortoiliac aneurysms (Wong et al. 2013). Despite reports of successful outcomes from off-label use of IBD for IIA aneurysms, further investigations should be conducted before it is approved for general use.

Four cases of technical failure were associated with incomplete embolization of IIA outflow, consequently leading to the development of a type 2 endoleak. Such an outcome suggests that the technique for outflow embolization is a significant determinant of clinical outcome. Meticulous embolization of the distal IIA segment or its branches is required to prevent backflow into the aneurysm. The sites of outflow embolization were categorized according to the location of embolic materials relative to the bifurcation of the anterior and posterior trunks. It was presumed that placing embolic materials closer to the bifurcation decreases the likelihood of backflow through the distal branches. Statistical analysis revealed that embolic materials placed relatively distally in the IIA branches were associated with a higher incidence of type 2 endoleak than when they were placed closer to the bifurcation or in the distal segment of the IIA. Endoleak was associated with patent branches that originated proximally to the embolization site. In one patient, endoleak through a patent iliolumbar artery resulted in aneurysm rupture and consequent mortality.

The development of endoleak was not necessarily associated with sac expansion. The overall rate of endoleak was 31.3%, while sac expansion was seen in 12.5% of all cases. These results are in line with those from previous reports, and support the finding that endovascular treatment of IIA aneurysm is effective in preventing sac expansion (Rana et al. 2014; Chen et al. 2021; Chaer et al. 2008; Kliewer et al. 2019; Melki et al. 2001; Antoniou et al. 2011; Harris 2009). However, reflecting on the exceptional case of endoleak that resulted in mortality, the importance of surveillance using cross-sectional imaging should not be underestimated.

Re-intervention was performed in three of four patients with sac expansion. A case of type 2 endoleak located at the level of the abdominal aorta was successfully managed by transcatheter embolization. Meanwhile, re-intervention was unsuccessful in two cases: one in which the feeding artery could not be catheterized and another in which the type 2 endoleak was misinterpreted as a type 1 endoleak.

Re-intervention was also performed in one patient who developed limb graft occlusion after undergoing EVAR for an aortoiliac aneurysm. A recent multicenter study reported a limb graft occlusion rate of 5.9 percent in a retrospective cohort of 924 patients undergoing EVAR for abdominal aortic aneurysms (Bogdanovic et al. 2021; Cochennec et al. 2007). The rate is likely to be similar in patients undergoing EVAR for IIA aneurysms that coexist with aortoiliac aneurysms. The occluded limb was successfully recanalized by thrombectomy and stenting.

Complications resulting from endovascular treatment of IIA aneurysm included two cases of femoral artery pseudoaneurysm and three cases of buttock claudication. Both cases of pseudoaneurysm formation were successfully managed by either percutaneous thrombin injection or surgical repair of the femoral artery. Buttock claudication was documented in three patients after undergoing unilateral IIA embolization. While the risk of developing buttock claudication is relatively higher after bilateral IIA embolization, it has been reported to develop in over a quarter of patients after unilateral IIA embolization (Kliewer et al. 2019; Lin et al. 2009; Kouvelos et al. 2016; Bosanquet et al. 2017).

There are some limitations to this study. First, this was a retrospective study lacking a standard protocol for the management of IIA aneurysms, including the indication for treatment, technical details of the endovascular procedure, and strategy for follow-up. Second, statistical analyses were insufficiently powered due to the small sample size of the cohort. For example, the efficacy of different endovascular techniques could not be compared. Such limitation is not exclusive to this study and reflects the rarity of IIA aneurysms. The significance of findings from this study should be further investigated in larger cohorts.

## Conclusions

Endovascular treatment of IIA aneurysm is effective in preventing aneurysm expansion. Endoleak may result from technical failure and embolization in the distal branches of the IIA. Surveillance is mandatory to prevent aneurysm-related mortality.
